# Composite Based on Biphasic Calcium Phosphate (HA/β-TCP) and Nanocellulose from the Açaí Tegument

**DOI:** 10.3390/ma11112213

**Published:** 2018-11-08

**Authors:** Rachel M. B. Valentim, Sabina M. C. Andrade, Maria E. M. dos Santos, Aline C. Santos, Victor S. Pereira, Izael P. dos Santos, Carmen G. B. T. Dias, Marcos A. L. dos Reis

**Affiliations:** 1 Post-Graduation in Natural Resources Engineering of the Amazon—PRODERNA, Federal University of Pará, Belém, Pará 66075-110, Brazil; marcosallan@ufpa.br; 2 Federal Institute of Education, Science and Technology of Pará—IFPA, Campus Belém, Pará 66093-020, Brazil; sabina_memoria@yahoo.com.br; 3 Post-Graduation in Mechanical Engineering—PPGEM, Federal University of Pará, Belém, Pará 66075-110, Brazil; maria.elizaabeth@hotmail.com (M.E.M.d.S.); alinecorecha@yahoo.com.br (A.C.S.); victorpr18@outlook.com (V.S.P.); izaelmec@gmail.com (I.P.d.S.); cgbtd@ufpa.br (C.G.B.T.D.)

**Keywords:** hydroxyapatite, calcium triphosphate, lignocellulosics, nanocomposite

## Abstract

The use of lignocellulosic remnants of the açaí agro-business will benefit the environment with a precursor material for biomedical applications. Nanocellulose (NC) allows the biomimetic growth of biphasic ceramics on its surface, with characteristics compatible with bone tissue, including bioactive properties and biocompatibility. In this study, the composites were obtained from açaí tegument (*Euterpe Oleracea* Mart.) NC using acid hydrolysis. The characterization performed by scanning electron microscopy showed the characteristic crystals of hydroxyapatite (HA) and calcium triphosphate (β-TCP) based on the results of X-ray diffraction, with the peak at 22°, showing the NC nucleation of HA and peak at 17° showing tricalcium phosphate (β-TCP). Fourier transform infrared spectroscopy confirmed the presence of O-H at 3400 cm^−1^ and C-H at 2900 cm^−1^, which is characteristic of cellulose; peaks were also observed at 1609 cm^−1^, verifying the reduction in lignin content. Groups PO_4_^−3^ at approximately 1070 cm^−1^, P-OH at 910–1040 cm^−1^, and HCO_3_^−^ at 2450 cm^−1^ confirmed the formation of HA and β-TCP. The zeta potential had a range of −11 ± 23.8 mV related to particle size, which had a range of 164.2 × 10^−9^–4748 × 10^−9^ m.

## 1. Introduction

Calcium phosphates are ceramic compounds with biomedical applications as their constituents are involved in the formation of bones and teeth, and they have excellent biocompatibility, bioactivity, no toxicity, variable degradation rates, and osteoconductivity [1]. Among the various types of calcium phosphate, hydroxyapatite (HA) and tricalcium phosphate (β-TCP) stand out. Their combinations are called biphasic calcium phosphate ceramics and they make it possible to obtain a composite with improved properties.

HA is a bioceramic composed of (Ca)_10_(OH)_2_(PO4)_2_, which is an important material. Due to its calcium phosphate composition equivalent to bone tissue [2], it has been used in bone repair and filling [3,4,5,6], applied as a filling to assist in the regenerative effect [7], and used in various other applications, such as biomedical composites [8,9]. For example, Mg-HA demonstrates significant larvicidal action and activity that inhibits pathogenic bacteria [10]. HA can be obtained from fish [11], bovine, chickens, and goat bones [12]. It can also be obtained through biomimetic growth using sugarcane bagasse pulp [13], which contains ions that are important for its formation.

Tricalcium phosphates (TCP) can occur in four crystalline forms: β-TCP, α-TCP, α’-TCP, and γ-TCP. A general-use β-TCP ceramic was analyzed through biological tests and medical applications, and it was found to surpass other bioceramics [14]. It has favorable characteristics such as structural stability, good lifespan, biocompatibility, and a favorable balance between absorption and degradation [15,16,17].

HA/β-TCP are ceramic biomaterials tested as a capping agent because they do not impair the exposed human dental pulp and can be used successfully as a basic material in endodontic therapy [18,19].

Cellulose is a polymer of vegetable origin, and it has advantageous properties such as good mechanical strength, which make it a desirable source for biomaterial in a sustainable, economics-based future [20]. The OH groups are abundant in cellulose, with their affinity for inorganic/organic substances leading to the preparation of hybrid materials and expansion to new applications without polluting the environment [21]. It has gained prominence as a biodegradable, nanocellulose material of natural origin. It can be used as a nanomaterial with excellent properties [22], and it may even be used as a substitute for petroleum products.

**Figure 1:**
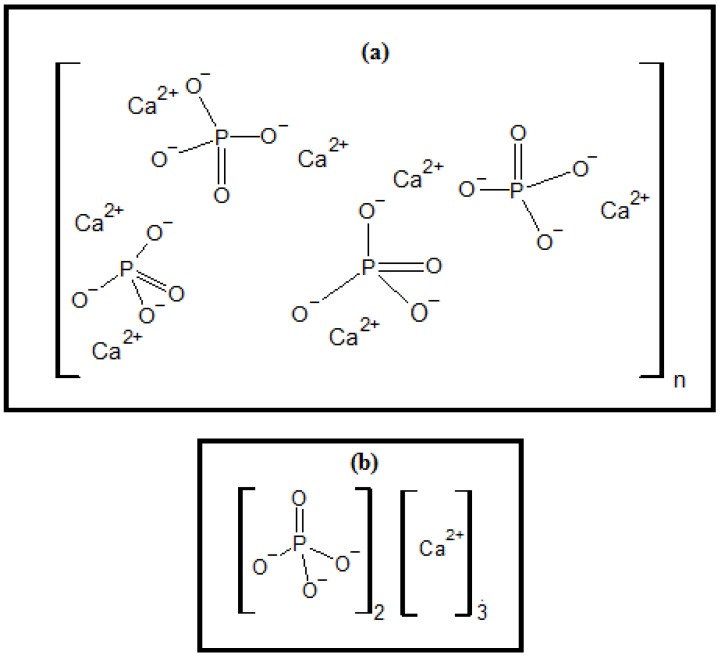

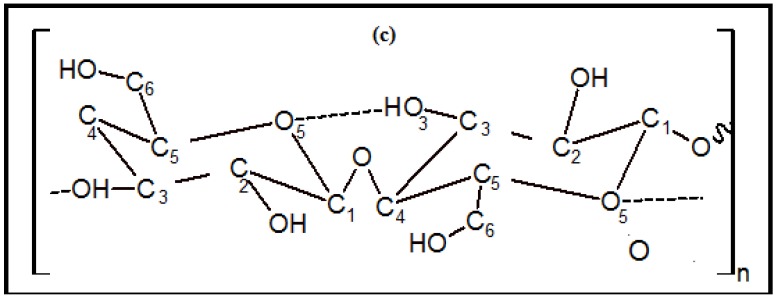
Chemical structure of (**a**) hydroxyapatite (HA); (**b**) tricyclic phosphate (β-TCP); and (**c**) cellulose.

In this study, a nanocomposite based on biphasic calcium phosphate (HA/β-TCP), shown in Figure 1a,b, was obtained by memetizing the nanocellulose extracted from the available and unexplored açaí tegument cellulose sources, as seen in Figure 1c [23]. It appears to be a promising material for use as a pulp-capping agent and for the treatment of dental caries as it comprises the same constituents of dentin, which better protects the pulp, resulting in benefits for oral health.

## 2. Experimental

### 2.1. Materials

The açaí core was collected in the agro-commercial sector of the city of Belém-PA and taken to the Eco-composites Laboratory at the Federal University of Pará (UFPA) to separate the parenchyma, monostelo and endocarp fractions. In four 30 min sessions, the endocarp was dried at 70 °C, processed using mill knives in three 2 h sessions, and passed through various sieves (#35, #42, #65, and #200) to obtain the tegument.

### 2.2. Method of Obtaining the NC/HA/β-TCP Nanocomposite

The açaí integument obtained from the endocarp was mercerized with sodium hydroxide (NaOH) at 80 °C and bleached with a 1 L solution containing 27 g of sodium hydroxide (NaOH), 75 mL of glacial acetic acid (C_2_H_4_O_2_), and 65 mL of hydrogen peroxide (H_2_O_2_) at 80 °C for 90 min. The nanocellulose (NC) was extracted using sulfuric acid (H_2_SO_4_) at 25 °C, which was a method adapted from Henrique [24]. The synthesis of HA and β-TCP on the NC surface through biomimetic growth occurred in an acid suspension, followed by neutralization using distilled water and evaporation by heating at 30–40 °C to obtain the powder.

### 2.3. Characterization of the NC/HA/β-TCP Nanocomposite

#### 2.3.1. Scanning Electron Microscopy (SEM)/Energy Dispersive Spectroscopy (EDS)

The morphological analysis of the tegument and the NC/HA/β-TCP was performed by SEM using the scanning electron microscope VEGA 3 LMU TESCAN, Brno, Czech Republic. Samples were placed directly on the 10 mm stubs with SPI Supplies 8 mm × 20 m double-sided carbon tape, followed by gold plating for 100 s; the samples were then analyzed at an acceleration voltage of 20 kV at a temperature of 25 °C. EDS was performed using a microanalysis system: model AZTec Energy X-Act, with resolution of 129 eV (brand name Oxford, High Wycombe, UK). The images were taken under a high vacuum.

#### 2.3.2. X-Ray Diffraction (XRD)

The crystalline structures of the samples were evaluated by XRD using a PHASER-BRUKER D2 diffractometer (Madison, SD, USA), operating at 30 kV and 10 mA, with Cu Kα radiation. The X’Pert HighScore Plus software 2.2d (2.2.4) version was used as a tool for the analysis of DRX standards by identifying the phase and performing crystallographic analysis.

#### 2.3.3. Fourier Transform Infrared (FTIR)

FTIR spectroscopic analyzes were performed using a Shimadzu^®^ IRPrestige-21 FTIR spectrometer (Tokyo, Japan). The spectra were recorded in the range of 4000 to 400 cm^−1^, with a resolution of 4 cm^−1^ and transmittance measurement mode. The pellets were prepared using a powdered sample and KBr (previously desiccated in an oven to a constant weight). The data were analyzed by IRsolution Optional software (original version).

#### 2.3.4. Measurement of Size and Zeta Potential

The surface charges and particle size were measured using a Zetasizer Nano series (Malvern Instrument, Royston, UK) from aliquots of the aqueous NC/HA/β-TCP suspensions under the following conditions: 0.8872 cP viscosity, a temperature of 25 °C, a particle refractive index of 1.59, a water refraction index of 1.33, and a particle absorption coefficient of 0.010. Three measurements were performed for each suspension.

## 3. Results and Discussion

### 3.1. NC/HA/β-TCP Nanocomposite Synthesis

NC/HA/β-TCP formation occurred immediately, 30 days, and 60 days after acid hydrolysis. The obtained colloidal material was powdered through evaporation of the water at 20–30 °C. These results are in agreement with Rodriguez-Lorenzo & Vallet-Register [25] because the synthesis of bioceramics can occur with variations in parameters such as precipitation and temperature, average pH, reaction time, reagent addition rate, and heat treatment temperature. The pH is an important factor in the morphology because when it is high, the solubility of the calcium is low, indicating saturation with nucleation and formation of small crystals. When it is low, the solubility of calcium is higher, presenting a low saturation. This means that it has a low nucleation frequency [26].

### 3.2. Scanning Electron Microscopy (SEM)

**Figure 2:**
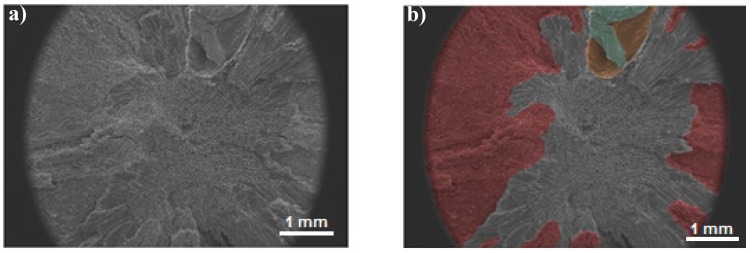

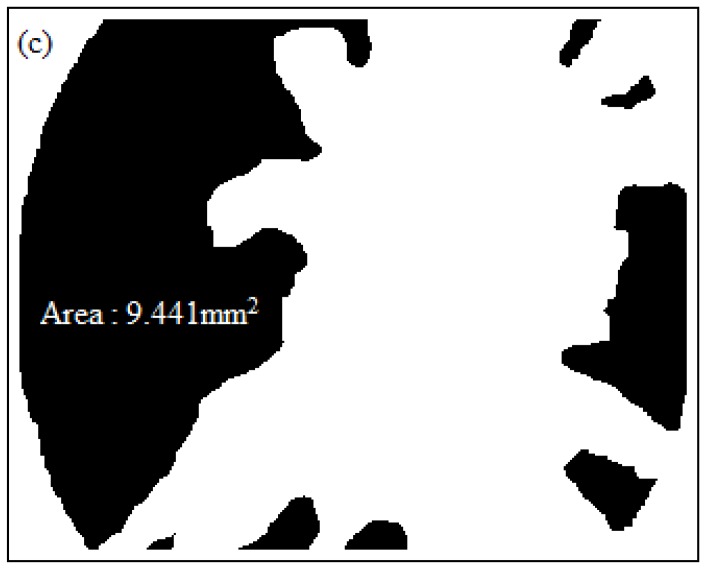
Tegument or shell is the outer part of the seed that acts against external factors. SEM micrographs of (**a**) açaí seed obtained by compression; (**b**) detachment of the açaí tegument by color (red color); and (**c**) measurement of the tegument area.

Figure 2 shows the surface of the compression fracture of an açaí seed in the SEM colored by the author, using the software program Adobe Photoshop (cc version), an image editor developed by Adobe Systems Incorporated, giving the embryo a green color inside its yellowish envelope and a red color at its tegument. The seed center with a cross-section of endosperm cells was not colored. For color subtraction, we used public domain software ImageJ, which has contrast processing functions.

**Figure 3:**
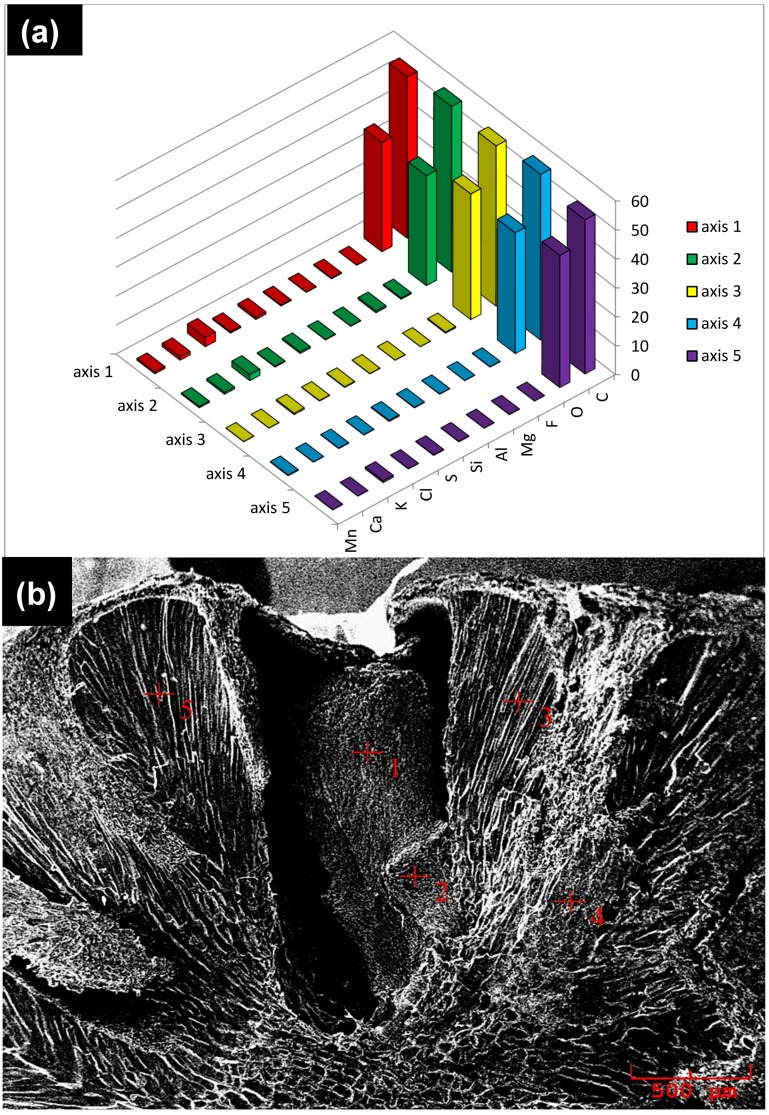
The Acaizeiro, Amazonian palm can occur in both mainland and varzea soils. EDS of mainland açaí palm seeds (**a**) and SEM of mainland açaí palm seeds (**b**).

**Figure 4:**
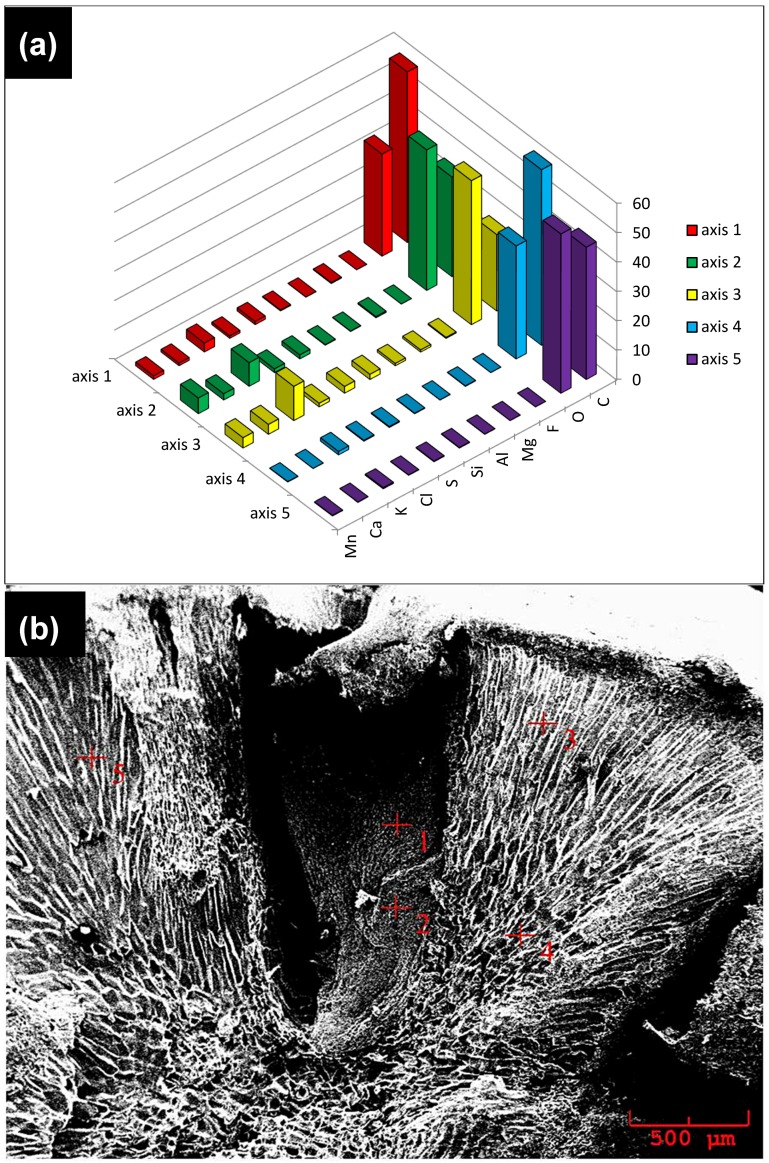
The Acaizeiro, Amazonian palm can occur in both mainland and varzea soils. EDS of the várzea soil açaí seed (**a**) and SEM of the várzea soil açaí seed (**b**).

Figure 3 shows the SEM/EDS results for the mainland seeds, and Figure 4 shows the results for the floodplain seeds. The results of SEM/EDS analysis indicate that there are higher concentrations of chemical components present in the analyzed varzea areas of the seeds—see Figure 3a,b and Figure 4a,b. The chemical elements mapped, red signals, in both seed types were the same: manganese (Mn), calcium (Ca), potassium (K), chlorine (Cl), sulfur (S), silica (Si), aluminum (Al), magnesium (Mg), fluorine (F), oxygen (O), and carbon (C). These results are important for the composition of biomaterials. They contribute to the formation of the HA/TCP nanocomposite as calcium phosphates have the capacity to replace cations and anions in their structure, which alters their crystallinity and solubility, acts as a regulating system of different ions in body fluids [1], and improves biological performance.

**Figure 5:**
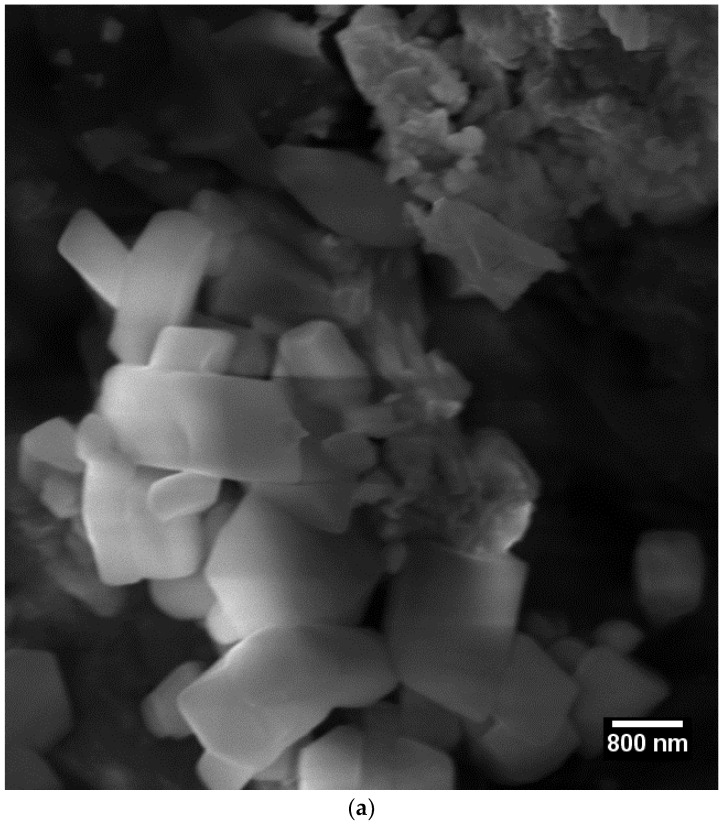

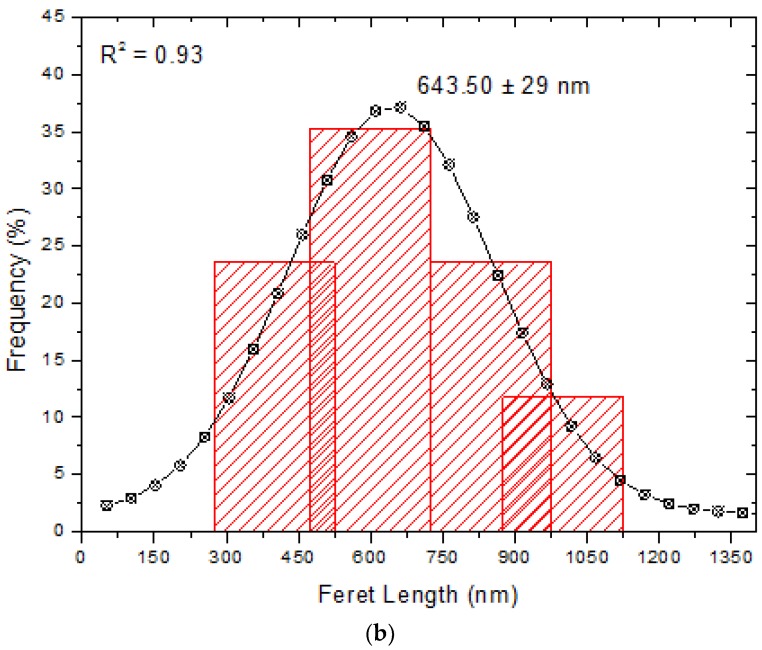
Analysis of the NC/HA/β-TCP surface and quantitative particle size. (**a**) SEM micrographs showing prismatic nanocellulose nucleating agent to obtain HA/TCP; (**b**) histogram relating to frequency and particle size.

The morphologies of the NC/HA/β-TCP nanocomposites were observed by SEM after extraction, 30 days, and 60 days later. In Figure 5a, the images reveal aggregate mineral crystals with varying sizes. The histogram, Figure 5b, relates to frequency and particle size, with a central peak of 643.50 nm representing the most common value, this is in agreement with the result of the Tyndall effect since the red laser has a wavelength around 660 nm, presenting a prismatic structure [27] and showing that the growth of HA/β-TCP crystals successfully occurred on the surface of the cellulosic material. It is observed that there is a relation between the hydrolyzing time and growth of the crystals, with a greater amount of time resulting in a greater quantity of crystals. According to Samir et al. [28], the acid hydrolysis destroys the amorphous regions, while the crystalline segments of the cellulose remain intact because the kinetics of the hydrolysis of the amorphous region is faster than that of the crystalline region as a result of the greater permeability of the amorphous region.

### 3.3. X-Ray Diffraction (XRD)

**Figure 6:**
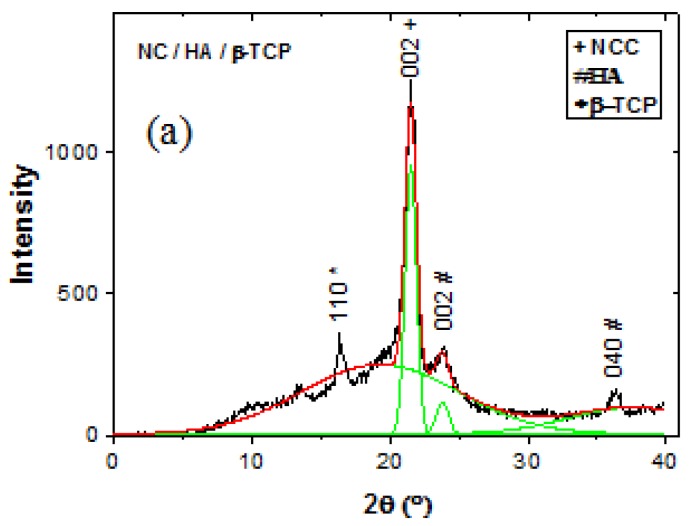

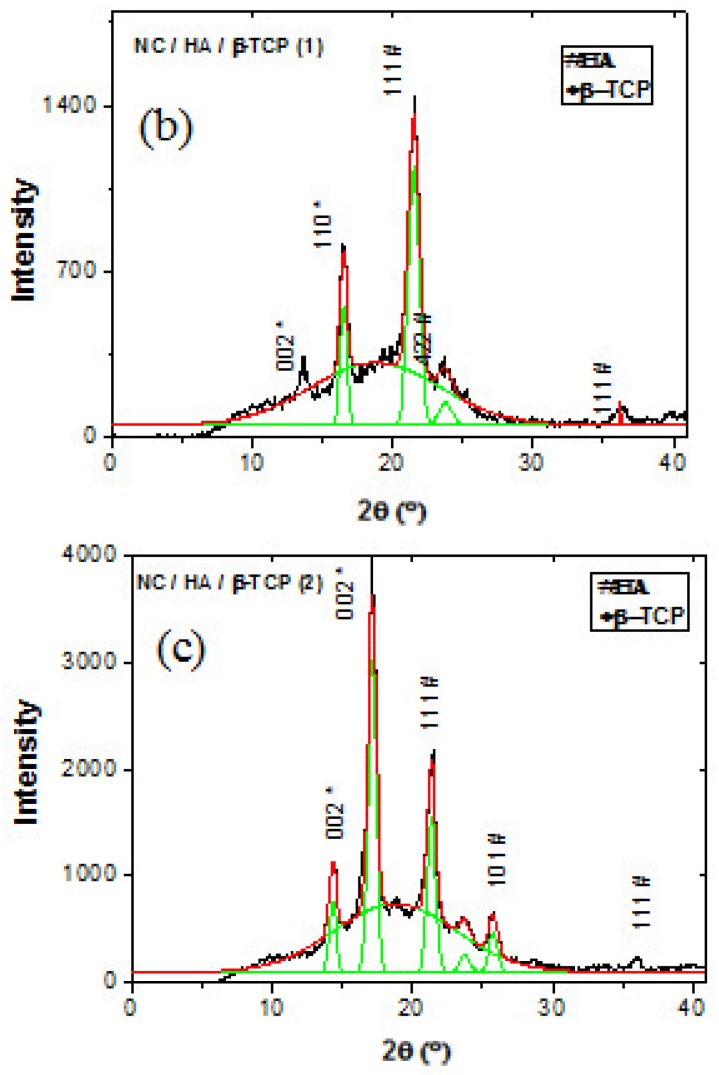
The composite powder structure was determined by XRD after extraction in three stages with different hydrolysis times: (**a**) 0 days; (**b**) 30 days, and (**c**) 60 days.

The XRD diffractograms (Figure 6a) show the nucleation of HA on the NC surface at 22°. Figure 6b,c show the characteristic peaks of tricalcium phosphate (β-TCP) and HA at 17° and 22°, respectively, which were nucleated on the type II cellulose surface. These results are in agreement with those of other authors [29,30,31,32].

### 3.4. FTIR Spectrum

**Figure 7:**
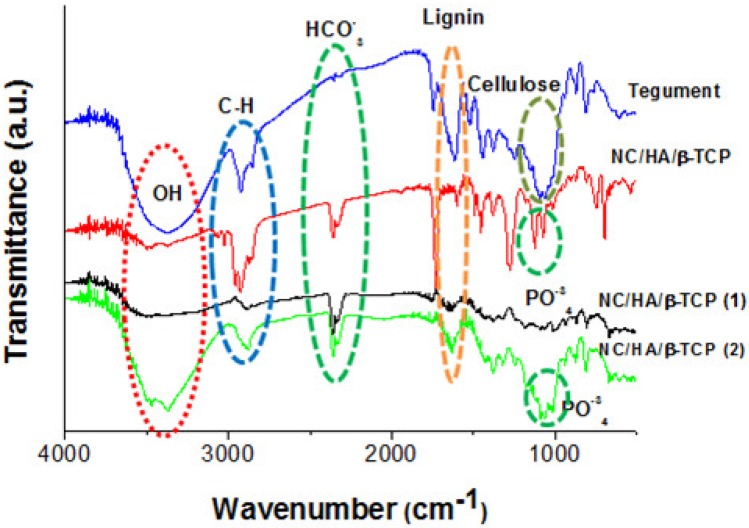
FTIR analysis of the source material (tegument) and the material obtained after extraction in three stages. FTIR obtained from tegument, NC/HA/β-TCP (immediately after hydrolysis), NC/HA/β-TCP (1) (30 days of hydrolysis), and NC/HA/β-TCP (2) (60 days of hydrolysis).

The FTIR spectrum (Figure 7) shows the characteristic O-H stretching vibration peaks at 3400 cm^−1^, C-H at 2900 cm^−1^, C-O at 1060 cm^−1^, and C-H glycosidic deformation at 897 cm^−1^ [33,34], which can be attributed to cellulose. This can be confirmed at approximately 1015, 1016, and 1027 cm^−1^, indicating C-O stretching [35]. An absorption band observed at 1056 cm^−1^ can be attributed to the stretching vibrations of the C-O-C bond [36]. The aromatic stretching of C=C with a strong C-C bond conjugated at 1609 cm^−1^ can be attributed to lignin [37], which shows a decrease in content during the process observed in the spectrum. The characteristic peaks of HA appear, with PO4^−3^ at around 1070 cm^−1^ in the vibration of (ν3) [38], P-OH at 910–1040 cm^-1^ [39], and HCO_3_^−^ at 2450 cm^−1^, showing the formation of NC/HA/β-TCP [40].

### 3.5. Measurement of Size and Zeta Potential

**Figure 8:**
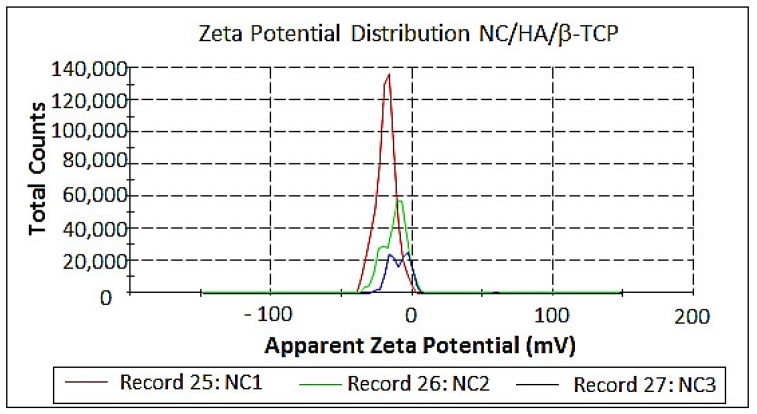

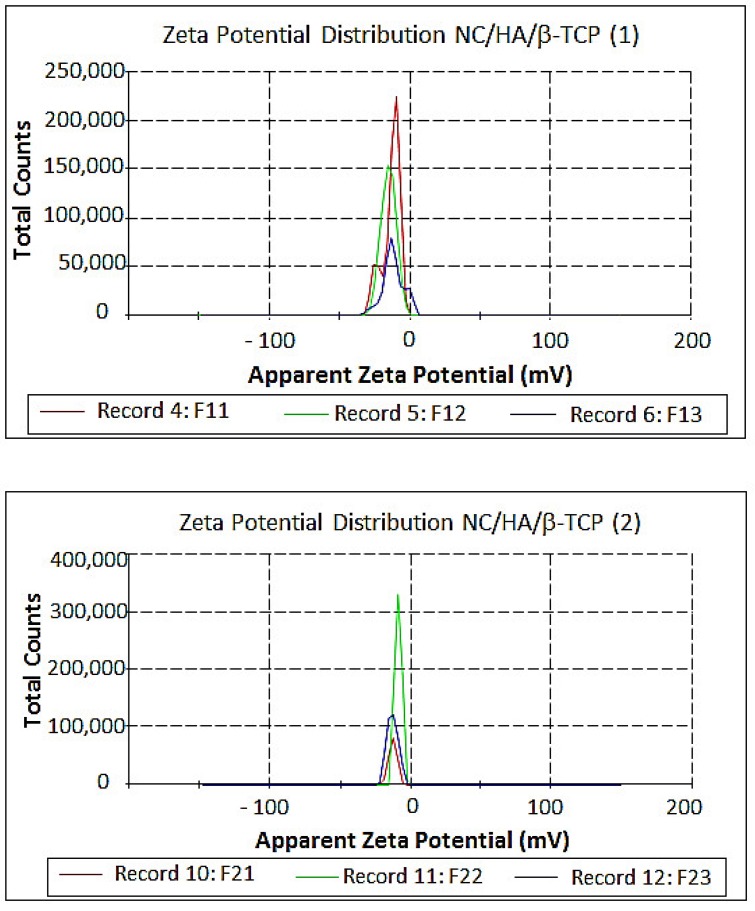
Zeta potential of NC/HA/β-TCP (immediately after hydrolysis), NC/HA/β-TCP (1) (30 days of hydrolysis) and NC/HA/β-TCP (2) (60 days of hydrolysis).

The zeta potential (Figure 8) measured the stability of the particles on their surface. The results are presented in Table S1 of the Supplementary Material and are shown to be insipient according to Morais et al. [41] and Jiang and Hsieh [33], showing little or no repulsive force because a greater zeta potential is associated with a greater electrostatic repulsion between particles [42].

**Figure 9:**
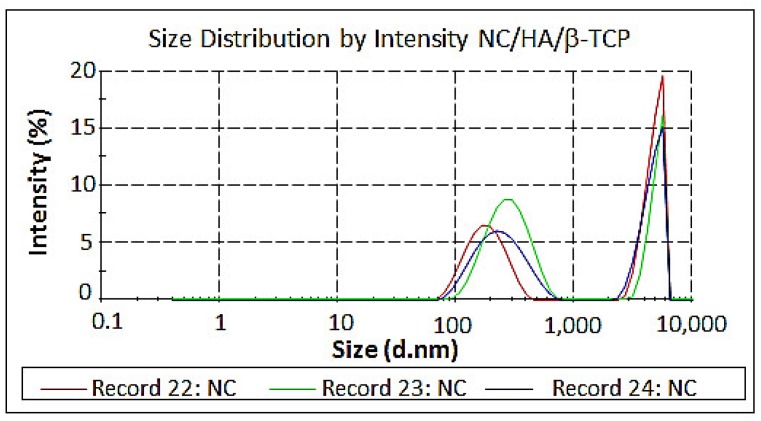

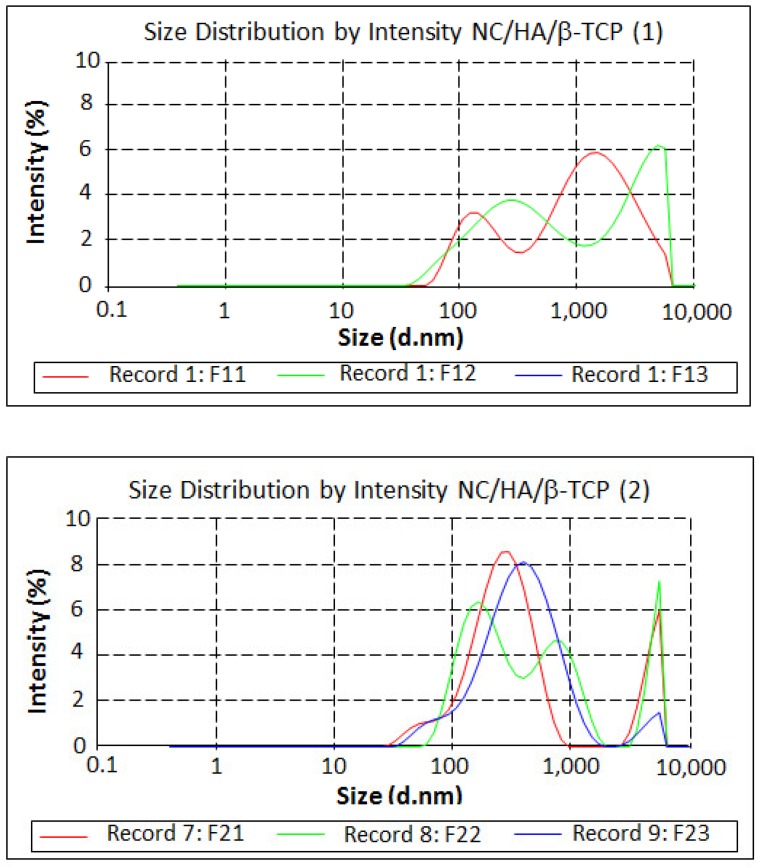
Size distributions of NC/HA/β-TCP (immediately after hydrolysis), NC/HA/β-TCP (1) (30 days of hydrolysis), and NC/HA/β-TCP (2) (60 days of hydrolysis).

The particle size distributions (Figure 9) were found using three records for each of the samples and are listed in Table S2 of the Supplementary Material.

The particle size results are in the gauge dimension, with a range of 164.2 × 10^−9^–4748 × 10^−9^ m, and they are related to the zeta potential results, which have an unstable range of −11 ± 23.8 mV with particle aggregation according to Zarbin [43,44,45].

The dispersed particles with a mean diameter in the range of 1nm to 1000 nm [46] and light scattering from the Tyndall effect form a colloidal system because the particles are too small to be identified by the naked eye, but their size is larger than that of the visible light wavelength [47].

## 4. Conclusions

The nanocellulose obtained from açaí integument (*Euterpe Oleracea* Mart.) was used for the biomimetic growth of biphasic ceramics (HA/β-TCP), which were used to form nanocomposites. These were characterized by SEM measurements and showed a prismatic trend that was confirmed by XRD and FTIR analyses, with the characteristic peaks of NC, HA, and β-TCP. The particle size had a gauge range of 164.2 × 10^−9^–4748 × 10^−9^ m, as evidenced by the zeta potential exhibiting instability, with tendencies to flocculation. These characteristics suggest the potential application of a biomaterial for the regeneration of bone tissue.

## Supplementary Materials

The following are available online at http://www.mdpi.com/1996-1944/11/11/2213/s1, Table S1: Zeta Potential Results, Table S2: Particle Size Results.
