# Supplementary material for: Composite Based on Biphasic Calcium Phosphate (HA/β-TCP) and Nanocellulose from the Açaí Tegument
Source: Materials (Basel). 2018 Nov 8;11(11):2213. doi: 10.3390/ma11112213 (PMC6266682; doi:10.3390/ma11112213)
Supplement: Supplementary file 1 [file materials-11-02213-s001.pdf]

materials

Article

Composite Based on Biphasic Calcium Phosphate (HA/β-TCP) and Nanocellulose from the Açaí Tegument

Rachel M. B. Valentim [a,*], Maria E. M. dos Santos [b], Aline C. Santos [b], Victor S. Pereira [b], Izael P. dos Santos [b], Sabina M. C. Andrade [c], Carmen G. B. T. Dias [b] and Marcos A. L. dos Reis [a]

[a] Post-Graduation in Natural Resources Engineering of the Amazon–PRODERNA, Federal University of Pará, Belém, PA 66075-110, Brazil; marcosallan@ufpa.br
[b] Post-Graduation in Mechanical Engineering–PPGEM, Federal University of Pará, Belém, PA 66075-110, Brazil; maria.elizaabeth@hotmail.com (M.S.); alinecorecha@yahoo.com.br (A.S.); victorpr18@outlook.com (V.P.); izaelmec@gmail.com (I.S.); cgbtd@ufpa.br (C.D.)
[c] Federal Institute of Education, Science and Technology of Pará–IFPA, Campus Belém, PA 66093-020, Brazil; sabina_memoria@yahoo.com.br
* Correspondence: rachelbarreira@yahoo.com.br

The zeta potential measured the stability of the particles on its surface and presents results in Table S1.

Table S1. Zeta Potential Results.

Sample	Average (mV)	Area (%)	Standard Deviation (mV)
NC/HA/ β-TCP	−18.8	100.0	6.98
NC/HA/β-TCP (1)	−11.2	80.1	3.83
NC/HA/β-TCP (1)	−23.8	19.9	3.17
NC/HA/β-TCP (1)	0.00	0.0	0.00
NC/HA/β-TCP (2)	−13.1	100.0	3.22
NC/HA/β-TCP (2)	0.00	0.0	0.00
NC/HA/β-TCP (2)	0.00	0.0	0.00

Table S2 shows the particle size distributions that were found using three records for each of the samples.

Table S2. Particle Size Results.

Sample	Peak	Size (nm)	Intensity (%)	Standard Deviation (nm)
NC/HA/β-TCP	1	4710	55.7	778.3
NC/HA/β-TCP	2	185.9	44.3	66.81
NC/HA/β-TCP	3	0.000	0.0	0.000
NC/HA/β-TCP (1)	1	1763	73.9	1196
NC/HA/β-TCP (1)	2	164.2	26.1	74.04
NC/HA/β-TCP (1)	3	0.000	0.0	0.000
NC/HA/β-TCP (2)	1	275.6	83.6	149.78
NC/HA/β-TCP (2)	2	4748	16.4	759.3
NC/HA/β-TCP (2)	3	0.000	0.0	0.000

© 2018 by the authors. Submitted for possible open access publication under the terms and conditions of the Creative Commons Attribution (CC BY) license (http://creativecommons.org/licenses/by/4.0/).
